# Does presenting perpetrator and innocent suspect faces from different facial angles influence the susceptibility of eyewitness memory? An investigation into the misinformation effect and eyewitness misidentification

**DOI:** 10.3389/fpsyg.2023.1213996

**Published:** 2024-03-28

**Authors:** Kara Deering, Melissa F. Colloff, Tia C. Bennett, Heather D. Flowe

**Affiliations:** Applied Memory Lab, School of Psychology, University of Birmingham, Birmingham, United Kingdom

**Keywords:** misinformation effect, eyewitness misidentification, eyewitness accuracy, eyewitness susceptibility, facial angles

## Abstract

**Introduction:**

This study investigated the effects of face angle congruency across stages of a misinformation paradigm on lineup discrimination accuracy.

**Methods:**

In a between-subjects design, participants viewed a mock crime with the perpetrator’s face from the front or profile angle. They then read a news report featuring an innocent suspect’s image from the same or different angle as the perpetrator had been shown. A subsequent lineup manipulated perpetrator presence and viewing angle of the lineup members, who were all shown either from the front or in profile.

**Results:**

No significant difference emerged in identification errors based on angle congruency between stages. However, accuracy was higher when faces were shown from the front angle, both during the initial event and the lineup, compared to the profile angle.

**Discussion:**

The results of this research underscore the importance of considering viewing angles in the construction of lineups.

## 1 Introduction

In June 1984, notorious serial killer Ted Bundy challenged his lineup identification process in the Court of Appeal, arguing that he was innocent, and that the witness identified him in error, solely because she had previously seen his picture in a newspaper story about the crime (Bundy, 1984). The prosecution countered that the newspaper image did not influence the witness’ memory because it showed Bundy’s face from the front, whereas the witness observed the perpetrator from a different angle, namely in profile view, during the crime. Supporting this, the witness stated that her identification was based on her initial memory of Bundy’s face from the profile angle. Further, the image of Bundy’s face she identified from the 10-image photographic lineup was also in profile view. The court dismissed Bundy’s appeal, implying the prosecution’s argument was more convincing. This study empirically examines the arguments put forward in this case, testing whether memory impairment arising from exposure to a suspect’s face depends on the congruence between the angle from which the perpetrator and the suspect are viewed by a witness.

From the defense’s perspective, whether the viewing angles of the perpetrator and newspaper suspect corresponded was immaterial. Rather, the witness’s post-event encounter with Bundy’s newspaper image, regardless of angle, altered her original memory of the perpetrator, leading her to misidentify Bundy, exemplifying a phenomenon known as the misinformation (MI) effect. The MI effect refers to a memory impairment that arises from exposure to misleading information about an earlier witnessed event that individuals subsequently integrate or substitute into their memory of the original event (Ayers and Reder, 1998).

Research has shown that eyewitness identification accuracy can be influenced by misleading post event information, including misleading face descriptions (Loftus and Greene, 1980) and composites (Topp-Manriquez et al., 2014; Sporer et al., 2020). The mechanism behind the incorporation of MI into the witness’s memory for original event has been widely researched and the subject of numerous debates. Some argue that misinformation overwrites or weakens the original memory traces (e.g., Loftus et al., 1978). Others have proposed that memory traces for the original and misleading information coexist, with interference (Bekerian and Bowers, 1983; Chandler, 1991) or source monitoring difficulties (Johnson et al., 1993) hindering accurate memory retrieval. Researchers have also extensively studied the boundary conditions of the effect, such as the whether the source of the misinformation is authoritative (Zaragoza et al., 2007).

Poorly encoded event details have been reported to be particularly susceptible to the influence of misleading information (Loftus and Greene, 1980). This susceptibility may be especially notable when the encoding involves a profile view of a perpetrator’s face. Key facial features such as the eyes, nose, and mouth, critical for accurate facial identification, are less visible from a profile angle (McKelvie, 1976; Fraser et al., 1990). This observation, combined with the holistic nature of face processing (Taubert et al., 2011), may lead to incomplete face encoding from profile views. Recent studies support this claim, indicating a decrease in lineup discrimination accuracy when witnesses encode a perpetrator’s face in profile rather than from a frontal view (Colloff et al., 2021). Consequently, a witness may be more susceptible to misleading post-event information when the perpetrator’s face is encoded solely from a profile angle, a hypothesis that we will refer to hereafter as the *encoding strength hypothesis*.

The impact of the angle of face presentation extends beyond the encoding phase to post-event information processing. The similarity between the original event and misleading information significantly contributes to the misinformation effect (Loftus, 1977). For instance, witnesses are more likely to incorporate post-event information into their memories when it is similar in nature to the original event, as demonstrated by the impact of shared contextual information on false memory formation (Carpenter et al., 2022). In line with the prosecution’s argument, these results lead to the hypothesis that the misinformation effect is more likely when the intervening innocent suspect’s face is presented from the same angle as the perpetrator, a hypothesis that we will refer to hereafter as the *facial angle congruency hypothesis*.

In testing our hypotheses, it is important to control for the angle of the lineup faces at test, even though police lineups typically show the faces from the front. The encoding specificity principle posits that the overlap between the cues at learning and test impacts memory performance (Tulving and Thomson, 1973). Consistent with this, discrimination accuracy, defined as the witness’s ability to distinguish between guilty and innocent suspects, is higher when the angle of the lineup faces aligns with the encoding angle (Colloff et al., 2021). This alignment of cues across encoding and the lineup might reduce the size of the misinformation effect, particularly if the angle of the test faces matches the angle of the perpetrator’s face during the crime, as was the case for the witness who identified Bundy.

The misinformation stage itself is an integral part of the encoding process and therefore necessitates consideration of face angle. Both Campbell et al. (2007) and Yamashita (1996) have argued that a recognition test presented in a format like the misinformation leads to an increased misinformation effect. Therefore, this study also explores the impact of face angle congruence between the misinformation face and the lineup members on discrimination accuracy. Specifically, we explored the possibility that witnesses are more easily misled when the angle of the faces shown during the misinformation and test stages matches.

## 2 Method

Full ethical approval for the current research was granted by the University of Birmingham Ethics Committee.

### 2.1 Design

The current hypotheses and analysis plan were pre-registered on the Open Science Framework before data were collected. A factorial between-subjects design was used, where participants were randomly assigned to one of eight conditions: 2 (encoding and test view: front, profile) x 2 (misinformation suspect view: front, profile) x 2 (lineup type: target-absent, target-present). Target-absent (TA) lineups contained the misinformation suspect presented among five fillers. The target-present (TP) lineups contained the guilty culprit among five fillers. A between-subjects design was used to avoid learning effects. The misinformation suspect and guilty culprit were never presented in the same lineup, and suspect position in the lineup was randomized for each participant. The facial angle shown during the lineup (i.e., at test) always matched the facial position shown at encoding. Therefore, it was also possible to collapse across conditions such that participants either received congruent facial angles (front encoding, front misinformation suspect, front lineup (FFF); profile encoding, profile misinformation suspect, profile lineup (PPP)) or incongruent facial angles (front encoding, profile misinformation suspect, front lineup (FPF); profile encoding, front misinformation suspect, profile lineup (PFP)) information. Table 1 summarizes each condition and the attendant abbreviation.

**Table 1:** Table to show Front (F), Profile (P), Target-Present (TP) and Target-Absent (TA) experimental conditions.

Encoding facial position	Facial position of misinformation suspect	Test: lineup condition and facial position	Condition summary	Facial angle congruency	Total per condition
Front	Front	Front; Target-Present	FFF-TP	Congruent	258
Front	Front	Front; Target-Absent	FFF-TA	Congruent	269
Front	Profile	Front; Target-Present	FPF-TP	Incongruent	268
Front	Profile	Front; Target-Absent	FPF-TA	Incongruent	252
Profile	Profile	Profile; Target-Present	PPP-TP	Congruent	251
Profile	Profile	Profile; Target-Absent	PPP-TA	Congruent	251
Profile	Front	Profile; Target-Present	PFP-TP	Incongruent	251
Profile	Front	Profile; Target-Absent	PFP-TA	Incongruent	251

### 2.2 Participants

Our preregistered data collection stopping rule was 2,000 participants.^1^ The sample size was based on collapsing across conditions to answer the research questions. Using mean differences and standard deviations observed in Mickes et al. (2012) as a guide, a power analysis indicated that, with a minimum of 250 participants per between-subjects condition, power would exceed 80%. We determined the sample size needed for >80% power to detect significant misinformation effect within each lineup condition. A bespoke power calculation tool developed for eyewitness lineup procedures was used.^2^ The misinformation effect size was based on effect sizes from the literature (Longmore et al., 2008; Bülthoff et al., 2019; Colloff et al., 2021), and it was reframed in terms of possible condition pAUC ratios, and used a Bonferroni-corrected alpha level based on the number of comparisons to be made (i.e., alpha = 0.05/2). An initial 2,947 participants were recruited using Amazon Mechanical Turk; all of whom were in the United Kingdom or America and aged 18 years or older. Individuals who had previously taken part in studies using the same crime video or lineup photographs were prevented from taking part in this study. Participants were paid 35 cents for taking part in the study, which took approximately 5 minutes. Participants were excluded from the final analysis if they incorrectly answered the attention check question or stated they had experienced significant technical issues that prevented them from witnessing either video (total *N* excluded = 896).

The final sample was 2,051 participants (55% female, 44% male, 1% preferred not to say or stated “other”; 18–89 years old, *M* age = 38.63, *SD* age = 12.74; 71% White Caucasian, 9% Black or African American, 6% Hispanic or Latino or Spanish, 5% East Asian, 2% South Asian, <1% American Indian or Alaska Native, <1% Native Hawaiian or Other Pacific islander, 3% said other and 3% preferred not to say).

### 2.3 Materials

A traditional misinformation paradigm was used in this study. The traditional paradigm involves three stages: encoding or experiencing an event, being presented with misinformation about the event, and then being asked to recall information about the event (Loftus, 2005). The misinformation paradigm allows researchers to test how an individual takes an external suggestion and misattributes this to their own personal memory of an event (Zhu et al., 2013).

#### 2.3.1 Mock crime videos

The video stimuli presented at the encoding stage was a mock crime video from Colloff et al. (2021), lasting approximately 17 s, depicting a Caucasian male perpetrator, approximately 30 years old, stealing a handbag from a female victim. There were two videos: one video presented the perpetrator from frontal view and the other presented the perpetrator from profile view.

The video stimuli presented at the misinformation stage was a news report video containing a photograph of the misinformation suspect. The video lasted approximately 36 s and contained an auditory narrative and subtitles explaining that a suspect had been arrested in connection with a recent handbag theft in the area. Specifically, the news report explained that the suspect was apprehended after police reviewed CCTV footage of the crime and found that the culprit looked like a local resident. A picture of an innocent suspect’s face was then shown on screen, either from a front facing or profile view. The misinformation suspect was male, aged approximately 30 years, and was similar in appearance to the perpetrator in the encoding video. The misinformation suspect was chosen based on pre-existing data from Colloff et al. (2021). These data showed that amongst the six filler faces used in the target-absent condition in the study, the misinformation suspect chosen was considered the most similar in appearance to the perpetrator. Faces shown in the encoding stage and the misinformation stage were both displayed for a duration of 7 seconds.

#### 2.3.2 Lineups

For the final stage of the misinformation paradigm, participant memories were tested using a six-person simultaneous photo lineup procedure – this method is not used by policing in the United Kingdom (which instead uses nine-person sequential video lineups) (Police and Criminal Evidence Act 1984, Code D, 2017), but it is used in many countries worldwide, including the United States (Fitzgerald et al., 2021). The photos showed the lineup members from the shoulder upwards, and the materials have been successfully used in prior research (Colloff et al., 2021). In the target-present lineup conditions, the guilty suspect (i.e., the perpetrator presented in the mock crime video) was shown amongst five fillers. In the target-absent lineup conditions, the misinformation suspect (i.e., the innocent suspect presented in the news report) was shown amongst five fillers. In line with police guidelines, Colloff et al. (2021) selected fillers who had similar facial attributes to the perpetrator in the mock crime video such that the suspect did not stand out (Police and Criminal Evidence Act 1984, Code D, 2017; Technical Working Group for Eyewitness Evidence, 1999). Colloff et al. (2021) established through mock witness-testing that the lineups were fair.

**Figure 1:**
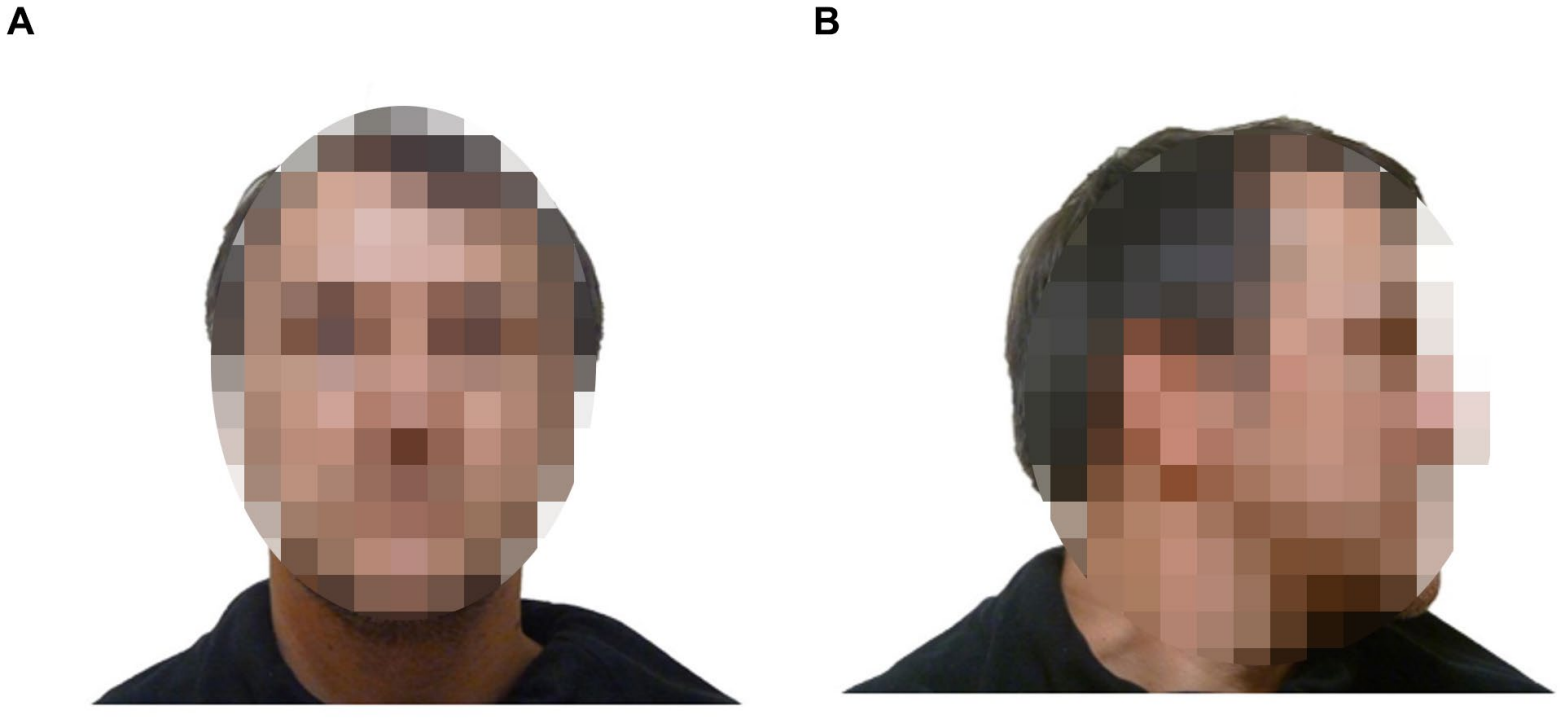
Guilty suspect lineup faces from the front **(A)** and right-profile **(B)**.

Lineups were presented with either right profile view or frontal facing lineup members (see Figure 1) that always matched the facial position presented to the participant at encoding. At present, there is a dearth of literature examining the effects of the different sides of the face on facial recognition performance. For example, some research has suggested that the right side of the human face has greater saliency as it bears more resemblance to the face as a whole (Gilbert and Bakan, 1973). On the other hand, Butler et al. (2005) found that when chimeric faces are used (where the left and right side of the face are combined from two different people), participants were more likely to bias their responses towards information on the left-hand side of the face. The current research did not use chimeric faces, it used photographs and videos of sole individuals. Therefore, the right profile faces were used in the “profile” conditions.

### 2.4 Procedure

Participants were initially provided with an on-screen participant information sheet that included information about the study and the participant’s right to withdraw. Participants were required to select “continue” on-screen to consent before they could take part. When they began the study, participants were asked several demographic questions (i.e., age, sex, and ethnicity/race).

All participants completed the three primary stages of the misinformation procedure: the encoding stage, the misinformation stage, and lineup test stage. First, in the encoding stage, participants were randomly assigned to watch one of two versions of the video: 1) the perpetrator’s face was shown in right profile view for the duration of the video, or 2) the perpetrator’s face was shown from the front, head on, for the duration of the video. After watching the video, participants completed a one-minute filler task consisting of anagram puzzles.

Next, the misinformation stage began. Participants watched the news report video and were randomly assigned to view the misinformation suspect either in the same pose as the mock crime video (front encoding, front MI; profile encoding, profile MI) or different pose (front encoding, profile MI; profile encoding, front MI). After viewing the news report video, participants then completed a further one-minute anagram filler task.

Finally, participants were presented with a simultaneous lineup test displayed in 2 rows of 3 photos. Participants were randomly assigned to view either a target-present or target-absent lineup. Before the lineup, participants were told that they needed to identify the person who they saw in the mock crime video. They were also informed that the guilty suspect may or may not be present in the lineup. Participants were asked to identify whether the guilty suspect was present, or to indicate “not present” if they believed the perpetrator was not present in the lineup. If a suspect was selected, participants were asked to indicate how confident they were in their identification response on a scale ranging from “guessing that he is the culprit” (50%) to “completely certain this is the culprit” (100%). If “not present” was selected, participants were presented with a forced choice lineup, comprising the same lineup members in the same position in the lineup as they had seen before, and asked to guess which suspect was the one they had seen in the crime video. They were then asked to indicate how confident they were that the person selected was *not* the person seen in the original crime video, on scale from “completely certain he is not the culprit” (−100%) to “guessing this is not the culprit” (−50%). This allowed for generating a “fullest possible” ROC curve that includes suspect IDs for the full range of the confidence scale (i.e., −100 to 100%). On completion of the confidence scale, participants were asked an attention check question (“How many people were in the first video you watched?”) and a technical check question [“Did you experience any technical issues when watching the mock-crime video (the first video) or the news report video (the second video)]. If “yes” was selected for the technical check question, participants were then asked to briefly explain the technical issue they had experienced. Participants who answered the attention check incorrectly, or who described experiencing significant technical issues (that prevented them from watching the videos), had their data excluded from final analysis. Upon completing these checks, participants were shown an on-screen debrief form which reiterated the details of the study, withdrawal procedures, and provided contact details for the researchers. Participants completed the study by closing the study tab on their computer.

## 3 Results

The number of subjects in each of the eight conditions is displayed in Table 1. Recall that when presented with the lineup at test, participants either selected a suspect from the six photographs presented (first lineup selection), or selected “Not Present,” which subsequently led to a second forced choice lineup. Response frequencies for the perpetrator, misinformation suspect, filler, and rejection (i.e., not present) decisions at each level of confidence for each condition are shown in Tables 2, 3 for first lineup selection and second forced choice lineup selection, respectively. The overall incorrect ID rate of the misinformation suspect (displayed in the proportion row in Table 2) is equal to the total number of misinformation suspect IDs from the target-absent lineups divided by the total number of target-absent lineups for each facial angle condition. Similarly, the overall correct ID rate of the guilty suspect (also displayed in the proportion row in Table 2) is equal to the total number of perpetrator IDs from target-present lineups divided by the total number of target-present lineups for each facial angle condition.

**Table 2:** Frequencies of perpetrator, misinformation suspect, and filler identification decisions by pose condition for first lineup respondents.

Confidence rating	FFF						PPP						FPF						PFP					
	Target-Present			Target-Absent			Target-Present			Target-Absent			Target-Present			Target-Absent			Target-Present			Target-Absent		
	Perp	Filler	Reject	MI	Filler	Reject	Perp	Filler	Reject	MI	Filler	Reject	Perp	Filler	Reject	MI	Filler	Reject	Perp	Filler	Reject	MI	Filler	Reject
100	82	2	–	5	2	–	36	2	–	45	0	–	94	0	–	3	1	–	46	0	–	31	0	–
90	63	1	–	8	5	–	51	3	–	27	1	–	58	1	–	6	5	–	46	1	–	32	3	–
80	28	2	–	12	4	–	32	1	–	18	3	–	27	1	–	8	5	–	33	6	–	24	1	–
70	12	4	–	2	1	–	17	1	–	17	0	–	19	2	–	8	4	–	17	0	–	21	1	–
60	10	0	–	3	5	–	13	3	–	14	3	–	10	3	–	9	1	–	19	2	–	11	1	–
50	3	1	–	2	0	–	5	1	–	4	0	–	3	2	–	1	1	–	3	1	–	5	2	–
Total	198	10	50	32	17	220	154	11	86	125	7	119	211	9	48	35	17	200	164	10	77	124	8	119
Proportion	0.77	0.04	0.19	0.12	0.06	0.82	0.61	0.04	0.34	0.50	0.03	0.47	0.79	0.03	0.18	0.14	0.07	0.79	0.65	0.04	0.31	0.49	0.03	0.47

**Table 3:** Frequencies of perpetrator, misinformation suspect, and filler identification decisions by pose condition for second forced choice lineup respondents.

Confidence rating	FFF				PPP				FPF				PFP			
	Target-Present		Target-Absent		Target-Present		Target-Absent		Target-Present		Target-Absent		Target-Present		Target-Absent	
	Perp	Filler	MI	Filler	Perp	Filler	MI	Filler	Perp	Filler	MI	Filler	Perp	Filler	MI	Filler
−50	10	3	4	8	18	2	3	4	12	2	7	11	12	5	16	4
−60	5	2	3	10	8	2	2	3	10	2	9	10	14	2	12	3
−70	4	2	10	11	12	3	14	3	1	4	13	10	9	1	10	2
−80	6	3	9	18	5	3	11	3	2	0	18	15	8	6	9	4
−90	3	3	13	28	8	4	22	9	7	1	20	17	7	3	13	8
−100	5	4	35	71	10	11	30	15	4	3	24	46	4	6	30	8
Total	33	17	74	146	61	25	82	37	36	12	91	109	54	23	90	29
Proportion	0.66	0.34	0.34	0.66	0.71	0.29	0.69	0.31	0.75	0.25	0.46	0.55	0.70	0.30	0.76	0.24

Each proportion is the proportion within each of the second forced choice lineups only. MI, misinformation suspect.

The overall ID rates of the suspect (TA lineups = misinformation suspect selection, TP lineups = guilty suspect selection) when a selection was made during the first lineup (Table 2) were FFF-TA = 0.12, FFF-TP = 0.77, PPP-TA = 0.50, PPP-TP = 0.61, FPF-TA = 0.14, FPF-TP = 0.79, PFP-TA = 0.49, PFP-TP = 0.65. For the second forced choice lineup (Table 3), the overall ID rates of the suspect were FFF-TA = 0.34, FFF-TP = 0.66, PPP-TA = 0.69, PPP-TP, 0.71 FPF-TA = 0.46, FPF-TP, 0.75, PFP-TA = 0.76. PFP-TP = 0.70. Further analyzes were conducted to explore these results, analyzing discrimination accuracy.

### 3.1 ROC analysis

Receiver Operating Characteristic (ROC) analysis (see Wixted and Mickes, 2015) was used to explore (1) the facial angle congruency hypothesis – that is, whether discrimination accuracy is higher when facial angles are incongruent across the misinformation paradigm (e.g., frontal encoding, profile misinformation, frontal test), and (2) the encoding strength hypothesis – that is, whether discrimination accuracy is higher when participants view a misinformation suspect from a profile facial angle at the misinformation stage when the encoding and test faces are frontal, compared to those who view the misinformation suspect’s face from a frontal angle when the guilty suspect’s face at the encoding stage and test faces are shown in profile.

In the current study, the ROC curves were created by plotting the hit rate (HR; the proportion of correct identifications of guilty suspects in TP lineups) against the false alarm rate (FAR; the proportion of incorrect identifications of misinformation suspects in TA lineups). Much previous lineup literature has plotted only positive IDs in ROC curves. Here, because participants in the study were forced to make an identification decision in the second forced choice lineup task, it was possible to extend the curves to contain negative IDs (second forced choice lineup selections). In order to plot the extended ROC curves, we took the six-point confidence scale from the first lineup selections (50%: guessing he is the culprit to 100%: certain he is the culprit) and the six-point confidence scale from the second, forced-choice lineup selections (−50%: guessing he is not the culprit to −100%: certain he is not the culprit) and combined them to create a single twelve-point scale (−100 to 100%). This followed a similar analysis procedure used by Colloff and Wixted (2020), where both partial and full ROCs were plotted. In both partial and full ROC analysis, the procedure with the ROC curve that falls furthest from the dashed line is the best at enhancing empirical discriminability (Colloff and Wixted, 2020).

To statistically compare ROC curves, pairwise comparisons between two conditions were made. To complete this pairwise comparison, the partial area under the curve (*p*AUC) was computed using the statistical package *p*ROC (Robin et al., 2011). The difference between the two *p*AUCs was then calculated and divided by the standard deviation of the difference estimated by bootstrapping, and therefore *D* is the measure of effect size. *D* is defined as $\frac{\left(\text{AUC}1-\text{AUC}2\right)}{S}$, where s is the standard error of the difference between the two AUCs estimated by the bootstrap method, with the number of bootstraps set to 10,000 (Mickes et al., 2012). In a *p*AUC analysis, the specificity cut-off must be set in the analysis. In each set of analyzes, a cut-off that was applied at the most liberal ROC point on the most conservative procedure.

As noted above, to increase the power of our analysis, “extended” ROCs were constructed that included both first lineup decisions (positive IDs where a face was selected) and second forced choice decisions (made after a negative “not present” decision), and the plan was to calculate the *p*AUC for the extended ROCs. However, when the extended ROCs were plotted, it was evident that the portion of the ROCs for the second forced choice lineup decisions were noisy. Previous research has found different results for positive and negative portions of ROCs (see Colloff et al., 2018; Colloff and Wixted, 2020). Therefore, for each research question, we plotted the extended ROCs (as we had initially planned), and also plotted the ROCs for the first lineup decisions only (i.e., the positive IDs, in the way that has typically been done in the lineup literature). For each research question, we present the *p*AUC results for extended ROCs that contain the positive and negative IDs (following our preregistered plan) and then the *p*AUC results for the positive IDs in the first lineups.

#### 3.1.1 Testing the facial angle congruency hypothesis

**Figure 2:**
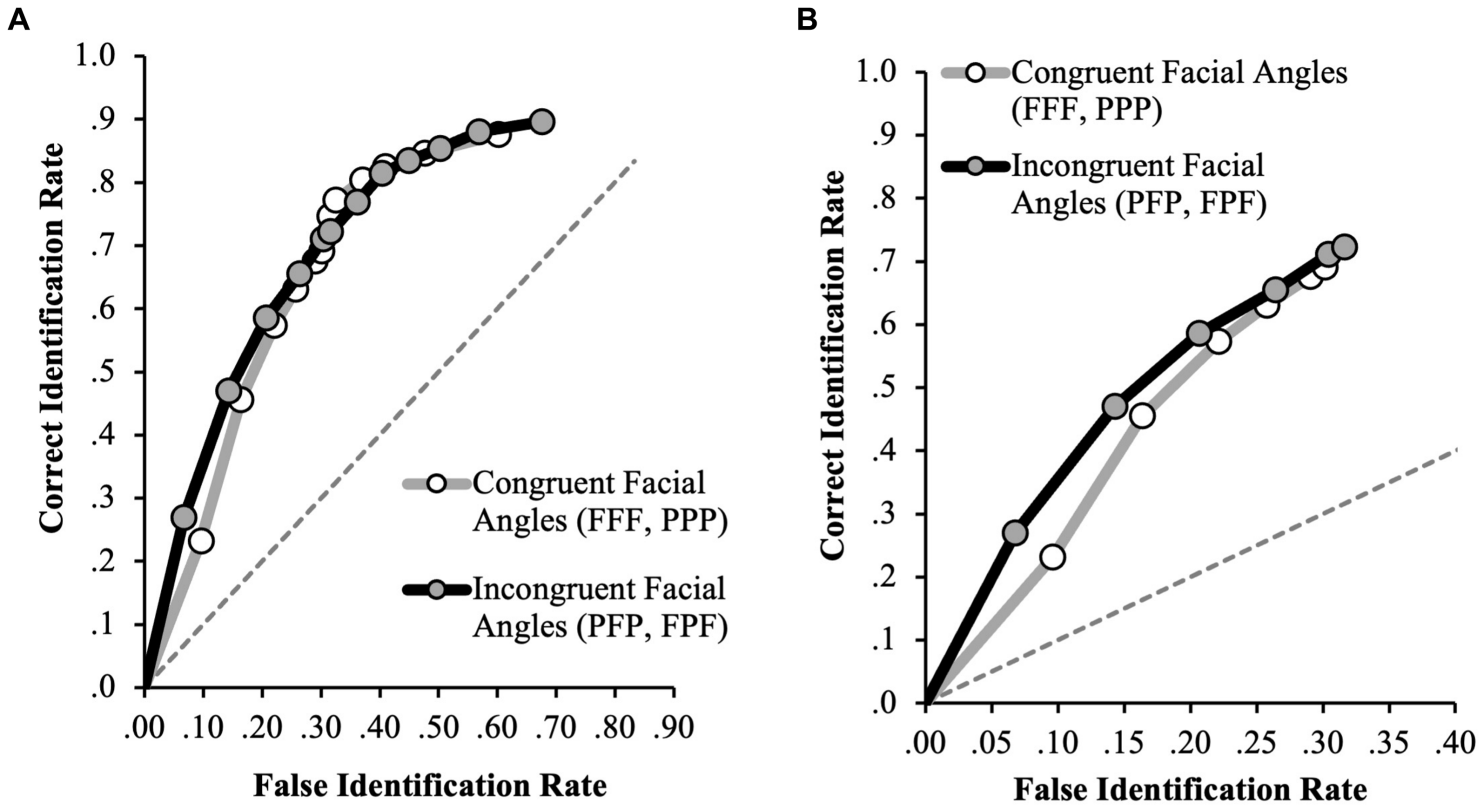
ROC data in the congruent facial angle (FFF, PPP) and incongruent facial angle (FPF, PFP) conditions for **(A)** positive IDs and negative ID decisions (extended ROCs) and **(B)** positive ID decisions only. The circular icons represent the empirical data. The dashed line indicates chance-level performance.

First, we investigated if discrimination accuracy differed depending on the congruency of facial angles. For the full ROC analyzes, the incongruent facial angle condition (FPF and PFP, *n* = 1,022) yielded a slightly higher pAUC (0.377, 95% CI [0.358–0.402]) than the congruent condition (FFF and PPP, *n* = 1,029) which was 0.362 (95% CI [0.336–0.387]). However, this difference was not statistically significant (*D* = 0.78, *p* = 0.44; specificity cut-off of 0.60, Figure 2A). Considering only the initial identification decisions, the incongruent condition yielded a slightly higher pAUC (0.131, 95% CI [0.114–0.148]) than the congruent condition (0.114, 95% CI [0.096–0.131]), yet this difference was also not statistically significant (*D* = 1.33, *p* = 0.19; specificity cut-off of 0.30, Figure 2B). Together, the results indicate that discrimination accuracy is similar regardless of facial angle congruency.

#### 3.1.2 Testing the encoding strength hypothesis

**Figure 3:**
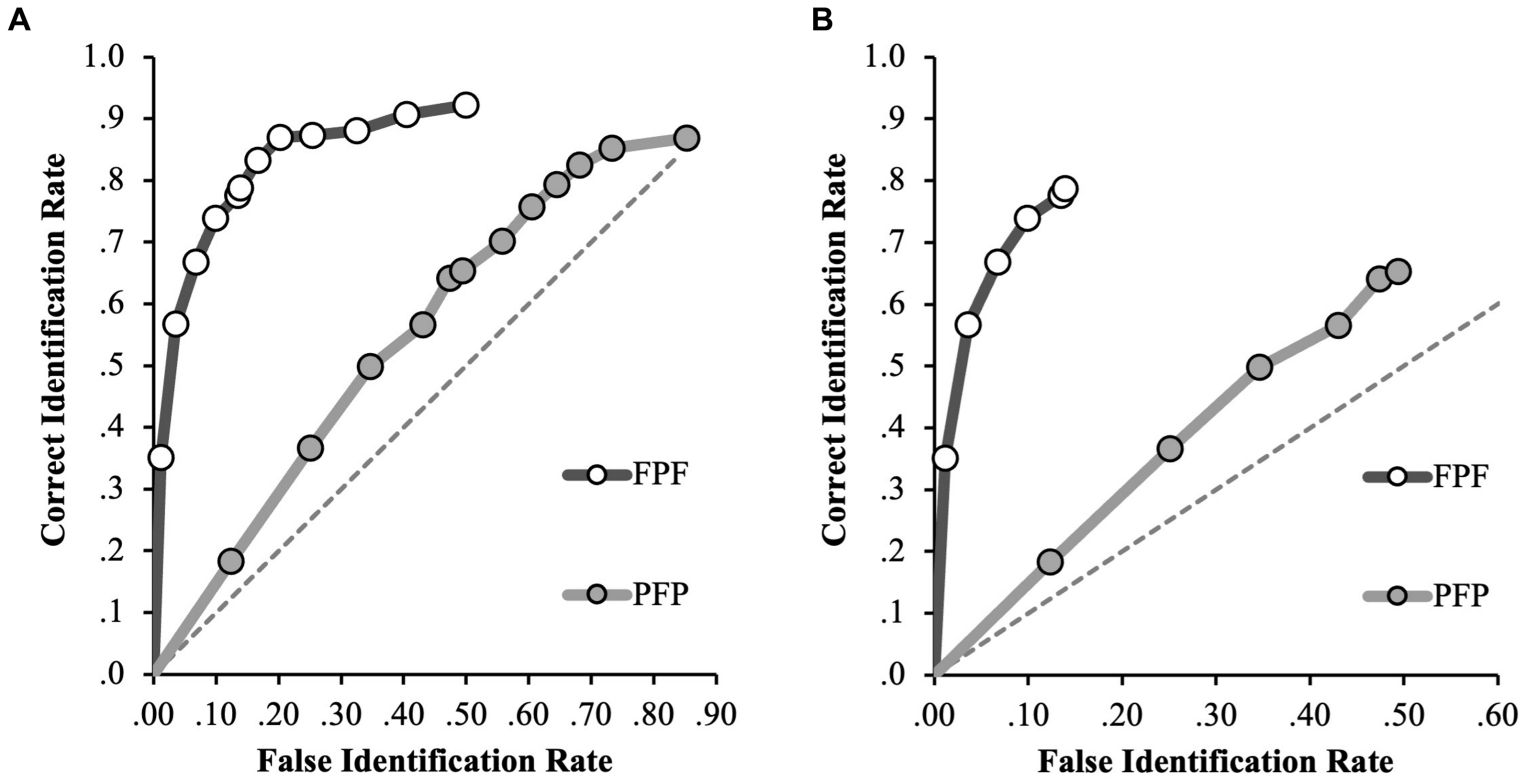
ROC data in the FPF and PFP conditions for **(A)** positive IDs and negative ID decisions (extended ROCs) and **(B)** positive ID decisions only. The circular icons represent the empirical data. The dashed line indicates chance-level performance.

Second, we investigated if encoding strength was stronger for frontal-view faces compared to profile-view faces. That is, whether participants are more likely to accept the misinformation (i.e., identify the misinformation suspect) when the perpetrator is presented from the profile view and misinformation presented from the front, compared to when the perpetrator is presented from the front and the misinformation is presented from the profile. To answer that question, we compared the ROC curves for the incongruent facial angle conditions – FPF and PFP (see Figure 3).

For the full ROC analyzes in the incongruent facial angle conditions (FPF and PFP), the FPF condition yielded a significantly higher pAUC (0.404, 95% CI [0.381–0.426]) than the PFP condition (0.176, 95% CI [0.143–0.209]), *D* = 10.97, *p* < 0.001 (specificity cut-off of 0.50, Figure 3A). This difference was also found considering only the initial identification decisions, where the pAUC for the FPF condition (0.101, 95% CI [0.088–0.112]) was significantly greater than the pAUC for the PFP condition (0.015, 95% CI [0.010–0.021]); *D* = 11.97, *p* < 0.001 (specificity cut-off of 0.14, Figure 3B). Therefore, for any false identification rate, the correct identification rate was increased by 129% in the FPF compared to the PFP condition when all identification decisions are considered and by 14.8% when only initial decisions are considered.

**Figure 4:**
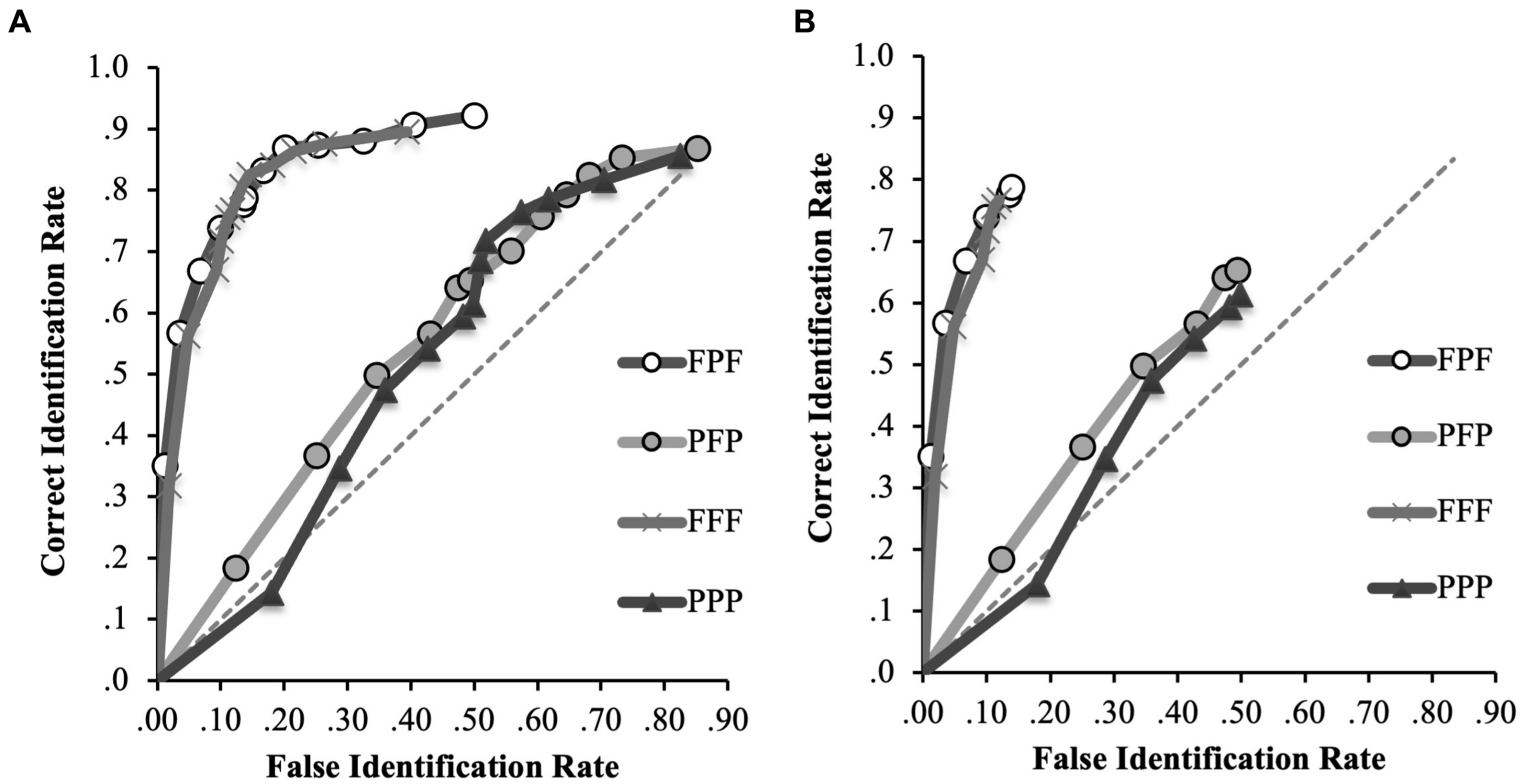
ROC data in the FFF, PPP, FPF and PFP conditions for **(A)** positive IDs and negative ID decisions (extended ROCs) and **(B)** positive ID decisions only. The circular icons represent the empirical data. The dashed line indicates chance-level performance.

To further explore the differences in discrimination accuracy between the incongruent conditions (i.e., FPF and PFP), ROC curves for every condition (FFF, PPP, FPF, PFP) were plotted on a single plot. Figure 4 shows the ROC curves for the FFF, PPP, FPF and PFP conditions.

In our evaluation of the full ROC curves across all conditions, several noteworthy patterns emerged (specificity cut-off of 0.39; Figure 4A). In the FFF condition, participants exhibited a pAUC of 0.297 (95% CI [0.275–0.317]), significantly outperforming those in the PPP condition (0.084, 95% CI [0.06–0.109]), *D* = 12.73, *p* < 0.001, and the PFP condition (0.110, 95% CI [0.086–0.138]), *D* = 11.39, *p* < 0.001. Therefore, for any false identification rate, the correct identification rate in the FFF condition increased by 253% compared to the PPP condition and by 170% compared to the PFP condition when first and second identification decisions are considered. The FPF condition (0.303, 95% CI [0.282–0.323]) also significantly surpassed the PPP condition, *D* = 13.48, *p* < 0.001, indicating that correct identifications for any possible false alarm rate increased by 175% in the FPF compared to the PPP condition. When comparing the FPF and PFP conditions directly, we found that the FPF condition had a significantly higher pAUC, *D* = 11.97, *p* < 0.001, indicating that correct identifications for any possible false alarm rate increased by 175% in the FPF compared to the PPP condition. However, we found no significant differences between the FFF and FPF conditions, *D* = 0.43, *p* = 0.67, or between the PPP and PFP conditions, *D* = 1.45, *p* = 0.15.

For completeness, the *p*AUC for the FPF and PFP conditions were calculated again for this analysis using the new specificity cut-off. Again, the *p*AUC for the FPF condition (0.303) was significantly higher than that for the PFP condition (0.110), *D* = 11.97, *p* < 0.001. This indicates that discrimination accuracy was significantly higher when participants were exposed to a frontal face at encoding and test compared to when they were exposed to a profile face at encoding and test. This suggests that the difference between the FPF and PFP in the previous analysis was due to a beneficial effect of viewing frontal faces at encoding and test, rather than a detrimental effect of viewing frontal faces at the misinformation stage.

Turning to the analysis of the initial identification decisions (specificity cut-off of 0.12; Figure 4B), we noted the following. The FPF condition (0.083, 95% CI [0.071–0.094]) significantly outperformed the FFF condition (0.063, 95% CI [0.051–0.076], *D* = 2.20, *p* = 0.01), indicating that correct identifications for any possible false alarm rate increased by 31.7% in the FPF compared to the FFF condition. However, the difference between the PFP (0.169, 95% CI [0.136–0.200]) and PPP (0.140, 95% CI [0.110–0.173]) conditions was not significant, *D* = 1.29, *p* = 0.20.

This suggests that discrimination accuracy was significantly higher when participants were exposed to the incongruent frontal encoding conditions (FPF) compared to the congruent frontal encoding conditions (FFF), but only for those who made IDs in the first lineup.

### 3.2 Confidence-accuracy characteristic (CAC) analysis

The relationship between confidence and accuracy was also explored in the current study. The link between high confidence ratings taken at the time of the identification and accurate lineup IDs has been well documented in recent research (Kebbell et al., 1996; Wixted et al., 2015; Wixted and Wells, 2017; Seale-Carlisle et al., 2019). Yet, there is a dearth of research looking at CACs for misinformation studies.

CAC analysis consists of plotting identification accuracy of suspect IDs (ignoring fillers IDs) for each level of confidence. For a six-person lineup procedure, CAC is given by;


$$CAC=\frac{\text{CIDconf}}{\text{CIDconf}+\text{FIDconf}}$$

CID*conf* is the number of correct guilty suspect IDs made with each level of confidence from target-present lineups. Alternatively, FID*conf* is the number of false IDs of misinformation suspects made with that same level of confidence from the target-absent lineups (Mickes, 2015; Seale-Carlisle et al., 2019). In this study, confidence ratings were binned into four levels of confidence: −100 to −80 and − 70 to −50 (for the forced-choice lineup decisions, or negative IDs), and 50–70 and 80–100 (for the first lineup decisions, or positive IDs). Unlike ROC analysis, the goal of CAC is to measure the relationship between confidence and accuracy (Mickes, 2015). As such, accuracy is plotted on the *y*-axis and confidence is plotted on the *x*-axis. This is useful from a practical standpoint, whereby the legal system is most interested in knowing the probability that a suspect who has been identified is actually guilty (Wilson et al., 2018).

**Figure 5:**
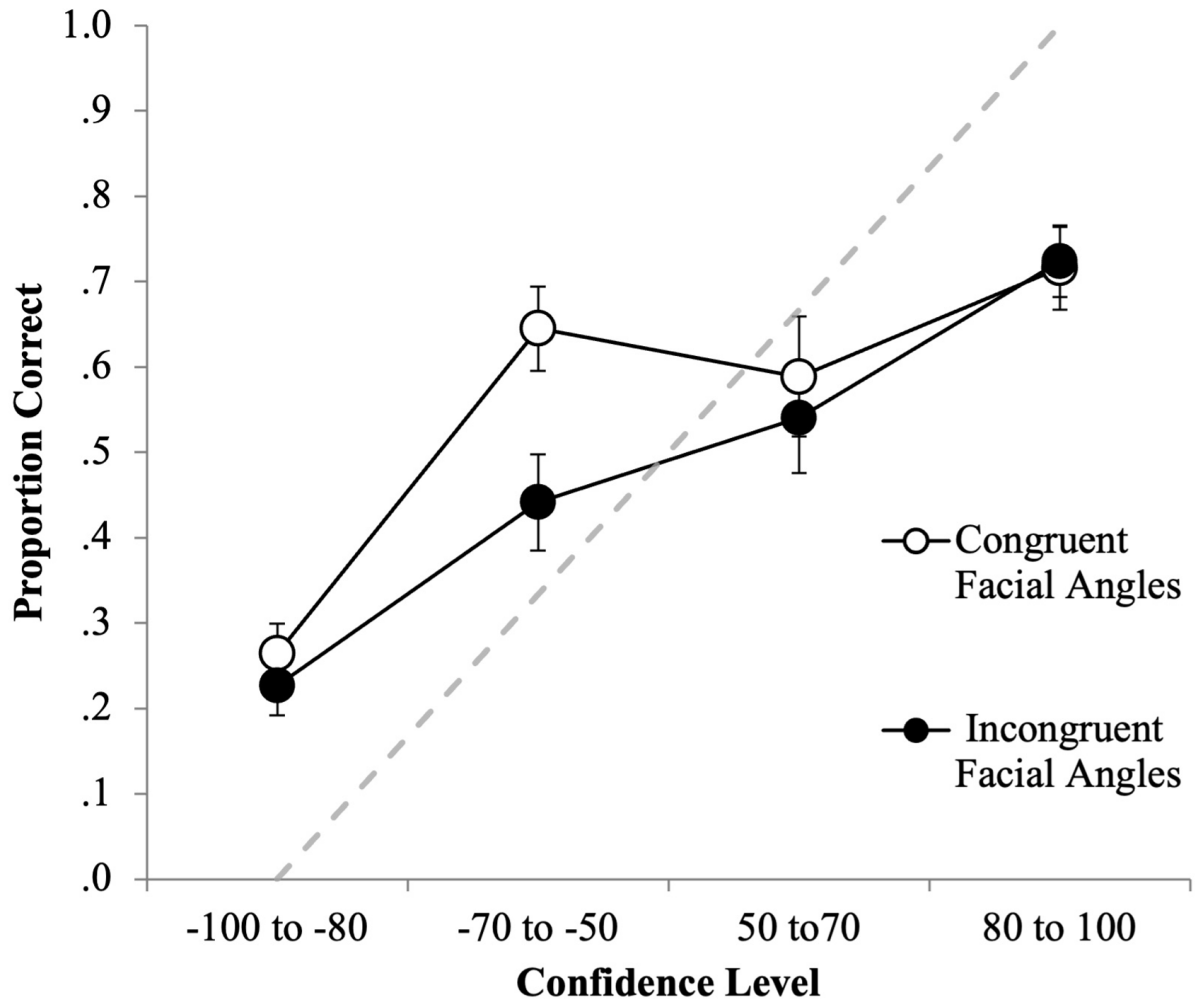
CAC data for the facial angle congruent (FFF, PPP) and incongruent (PFP, FPF) conditions for first- and second-line up decisions. The circular icons represent the empirical data. The dashed line indicates chance-level performance. The error bars also represent the standard error.

First, CAC curves were plotted for the congruent facial angle (FFF, PPP) and incongruent facial angle (FPF, PFP) conditions. Figure 5 shows that there appeared to be a relationship between confidence and ID accuracy in both conditions, because, generally speaking, as accuracy increased, so did confidence. However, the relationship was stronger in the incongruent facial angle conditions. In the congruent facial angle conditions, there was a relationship within the negative IDs (i.e., −70 to −50 yielded a higher proportion correct than −100 to −80) and within the positive IDs (i.e., 80 to 100 yielded a higher proportion correct than 50 to 70) but, for some reason, IDs made with a confidence rating of 50 to 70 were less accurate than those made with −70 to −50. For both conditions, it is important to note that high confidence did not indicate high accuracy, as participant were overconfident at high confidence. Participants who made 80–100% confidence judgments where only approximately 70% accurate in their suspect IDs. This is likely due to the deleterious effect of misinformation.

**Figure 6:**
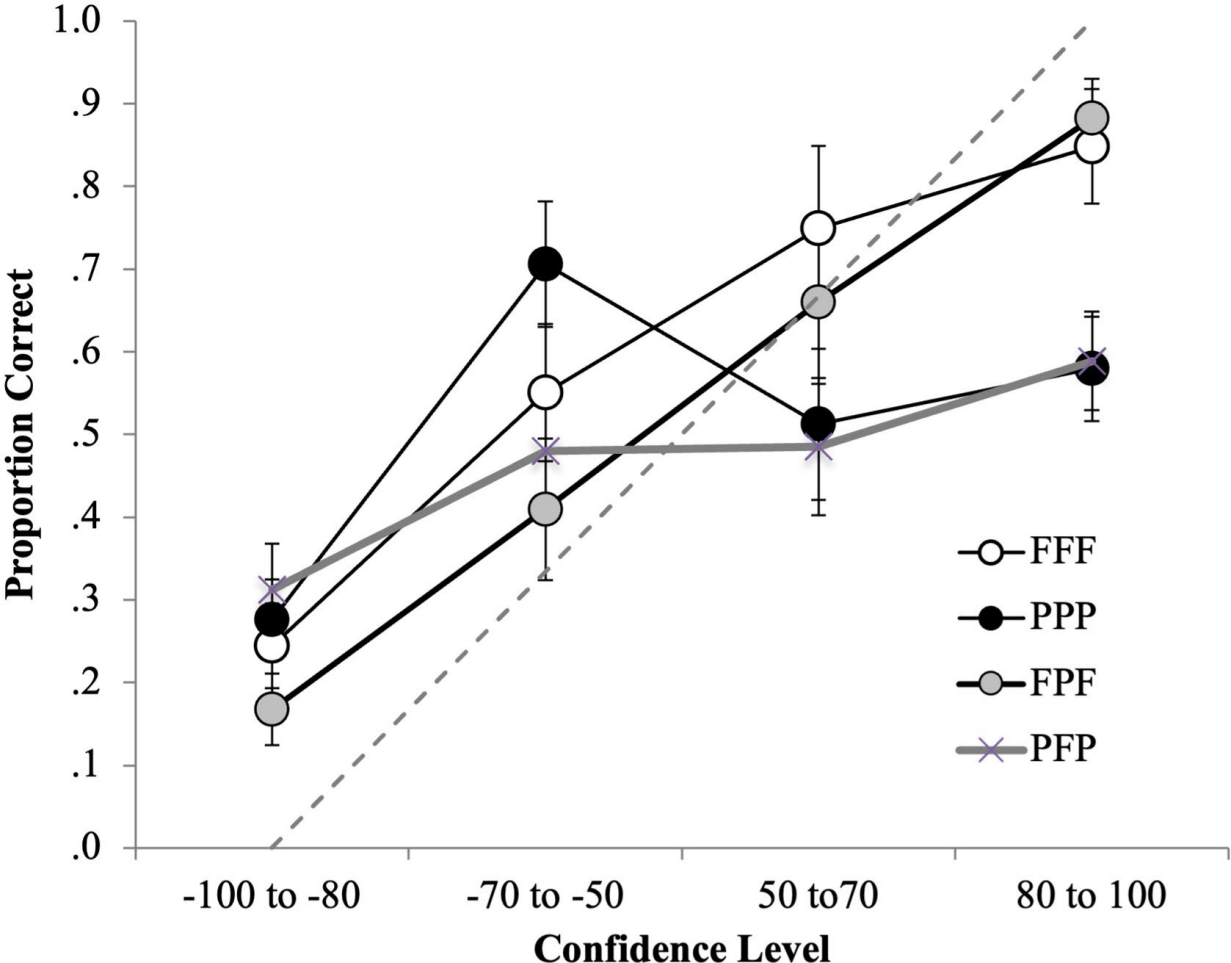
CAC data for the all four facial angle (FFF, PPP, FPF, PFP) conditions for first- and second-line up decisions. The circular icons represent the empirical data. The dashed line indicates chance-level performance.

To further explore the relationship between confidence and accuracy in the frontal and profile facial angle encoding conditions, all four conditions were plotted for the CAC analysis. Figure 6 shows the CAC analysis for the FFF, FPF, PPP and PFP conditions.

For the frontal encoding and test conditions (FFF and FPF) there was a relationship between confidence and accuracy, because as confidence increased, so did accuracy. IDs made with high confidence (i.e., 80–100% confidence rating) were also higher in accuracy (around 80% accurate) in the frontal facial angle encoding conditions compared to the profile facial angle encoding conditions. For the profile encoding conditions (PPP and PFP), there appeared to be a weaker relationship between confidence and accuracy. Moreover, participants were overconfident that they had identified the perpetrator when they provided high confidence ratings; they were only approximately 55% accurate when they were 80 to 100 confident.

## 4 Discussion

The current research explored the impact of facial angle on misinformation susceptibility. It was hypothesized that facial angle congruence between encoding/test and misinformation (e.g., FFF and PPP) would decrease discrimination accuracy compared to facial angle incongruence (e.g., FPF and PFP). The facial angle congruence hypothesis was not supported, as there was no significant difference between congruent and incongruent facial angle conditions. This suggests that participants were no more likely to be misinformed if the misinformation was more like encoding and test compared to when the misinformation was more different to encoding and test. One explanation for this could be that because the facial angle at encoding and test were always congruent, this may have had a stronger impact compared to congruent facial angles at misinformation and test stages. That is, matching the context at misinformation and test is less problematic for discrimination accuracy, so long as the test context remains the same as that experienced at encoding. This supports previous research by Bruce (1982), who found that when individuals learned a frontal face and were subsequently tested with a frontal face, they were able to recognize faces more accurately and quickly compared to when they were tested with faces posed a 45° angle (profile). Although the prediction was not met, a dearth of previous research has fully explored congruent and incongruent facial angles at different stages of the misinformation paradigm. Therefore, this finding has contributed to the growing understanding of facial angle manipulations in the misinformation paradigm.

Based on previous research regarding the strength of frontal face encoding, it was also hypothesized that front-view encoding would enable better discrimination accuracy compared to profile-view encoding. This hypothesis was supported because performance was generally better when the encoded face was front facing compared to profile. This suggests that frontal face encoding and test is superior in memory to profile face encoding and test.

An additional encoding strength hypothesis was considered, proposing that discrimination accuracy would be higher when participants were presented with a profile facing misinformation suspect when the encoding and test faces are frontal (FPF), compared to when participants view a frontal misinformation face when the encoding and test faces are profile (PFP). This hypothesis was supported, as discrimination accuracy was better in the FPF condition than the PFP condition. To explain this result, we initially proposed that discrimination accuracy may have been better in the FPF condition than the PFP condition due to the strength of the facial angle at the misinformation stage. Put another way, discrimination accuracy in the PFP condition may have been lower than the FPF condition due to the stronger encoding of the front facing misinformation, opposed to the profile facing encoding and test stages. Likewise, higher discrimination accuracy found in the FPF condition may be because profile misinformation would not have had the same encoding strength as the original front facing perpetrator, making it easier for participants to discriminate between faces. This would support previous research, whereby frontal faces have been considered to provide more information than a profile face (McKelvie, 1976), thus leaving a stronger memory trace (Fraser et al., 1990; Meltzer and Bartlett, 2019).

However, our further analyzes suggest this is not the case. When we compared all four facial angle conditions (FFF, PPP, FPF, PFP), further support for a front face encoding benefit was evident. That is, a frontal encoding benefit over profile encoding was observed in the FFF and FPF conditions compared to the PPP and PFP conditions. This difference cannot be explained by differences of facial angle at the misinformation stage, and instead must be explained by difference of facial angle at encoding (and test). Together, the findings support the encoding strength hypothesis and previous face memory literature, where frontal face encoding is argued to be superior to other poses (Colloff et al., 2021). This also supports the holistic encoding hypothesis, which suggests that instead of processing faces as a collection of separate, distinct, facial features, we instead process the face as a perceptual whole (Taubert et al., 2011). Therefore, seeing a criminal’s face from a frontal view at encoding and test means that participants can engage in holistic facial encoding and recognition. We also know that a frontal face provides more perceptual information than a profile face (Meltzer and Bartlett, 2019) and that this perceptual information can be beneficial for facial recognition.

For most of the findings, the ROC analysis of the positive lineup IDs (first lineup decisions) replicated the findings from the extended ROC analysis including negative IDs. However, when results for the partial positive portion of the curve were calculated for the FFF and FPF condition, discrimination accuracy was significantly higher in the FPF condition compared to the FFF condition (*p* = 0.03). This significant difference was not observed in the extended ROC analysis. A possible explanation for the significant finding is that the congruence between encoding, misinformation, and test in the FFF condition may have made it more difficult for participants to discriminate between the guilty suspect and the misinformation suspect than the FPF. This would, in part, support the proposed facial angle congruence hypothesis. But if that were true, it is not clear why the same pattern of results was not observed in the profile encoding conditions (i.e., no significant difference between PPP and PFP), or on the extended ROC. What we do know is that the analysis found significantly better discrimination accuracy in the frontal encoding conditions compared to the profile encoding conditions. One reason this finding may not have been observed in the profile encoding condition is due to the overall poor discrimination accuracy in the PPP and PFP conditions, where discrimination accuracy was only marginally better than chance. Moreover, other research has found the predicted pattern of results only in the positive IDs and not the negative IDs (see Colloff et al., 2018; Colloff and Wixted, 2020), but it is not yet clear why that is the case. Nevertheless, because this result was only found in front encoding conditions (i.e., FFF, FPF), but not profile encoding conditions (i.e., PPP, PFP), and was only observed in the positive ID portion of the ROC and not the extended ROC including negative IDs, the significant result should be interpreted with caution and further research is needed.

### 4.1 Practical implications

We found that the angle of the misinformation (congruent or incongruent with study and test) was not an important determinant of identification accuracy. Instead, we found that when the encoding face was presented from a profile view discrimination accuracy was significantly poorer than when the encoding face was presented from the front. The witness in the Ted Bundy case *did* encode Ted Bundy from the profile view. Whilst it is highly probable that she correctly identified Bundy (considering the abundance of evidence implicating him), the lower discrimination accuracy results for profile encoding in the current study are noteworthy. This underscores the importance of ensuring that the angle of the lineup faces matches the angle(s) shown during encoding. Previous research has found that discrimination accuracy for faces encoded in profile view is higher when the lineup faces are also presented in profile view (Colloff et al., 2021). Interestingly, the lineup the witness in the Bundy case viewed showed the lineup members also in profile view, providing cues that likely matched the encoding context and supported her memory retrieval.

Moreover, the results suggest that witnesses who have encoded perpetrators from profile view may be less reliable because they were found to have lower accuracy at high-confidence and have a poorer confidence-accuracy relationship than witnesses who have encoded perpetrators from the front. One explanation for this is that because the discrimination performance was so low in the PPP and PFP conditions (only marginally higher than chance), this impacted participant’s ability to assign appropriate confidence ratings. The poor confidence-accuracy relationship in the PPP and PFP conditions are consistent with findings from previous research that has found a poor confidence-accuracy relationship when memory accuracy is below chance (see Weber and Brewer, 2003; Nguyen et al., 2017). Theoretically, participants who are guessing should not be more confident in their guess that resulted in a correct identification than a guess that resulted in an incorrect identification (Nguyen et al., 2017). Furthermore, participants who are guessing (i.e., whose memory signal is weak) would have more relaxed criterion for identifying faces. Therefore, they are predicted to be less confident in their responses than participants who make recognition judgments based on more information in memory (i.e., stronger feelings of familiarity with a face). This suggests that accuracy is more likely to fluctuate around chance levels at lower levels of confidence.

Court systems may not always consider confidence when evaluating eyewitness IDs (Juslin et al., 1996). It can be argued that the reason for this is because confidence ratings are susceptible to influence. For example, other research has found that a poor correspondence between confidence and accuracy has also been associated with conformity to misinformation, whereby participants are misled but still provide high confidence ratings (Mudd and Govern, 2004; Foster et al., 2012; Spearing and Wade, 2021).

### 4.2 Limitations and future directions

In considering these findings, it is important to note a methodological limitation of the current research. Given that participants were always exposed to the same facial position at encoding and test, this research has not considered the potential influence that incongruent facial angles between encoding and test in the misinformation paradigm may have on misinformation susceptibility. Previous research suggests that people will be slower to recognize a face and less accurate in their recognition if the viewing angle of a face is changed (for example, between front facing and ¾ facing) between initial presentation and test compared to when it remains unchanged (Bruce, 1982). However, it is noted that this finding has not been explicitly explored in the misinformation paradigm. Likewise, the full impact of facial viewing angle manipulations across the three stages of the misinformation paradigm have not been explored in this single study. It will be important for future research to explore how further facial manipulations at test could impact misinformation susceptibility.

Similarly, the present study only included one suspect and one misinformation face, however, to counter any mediating factors that may be involved in eyewitness discrimination accuracy (for a discussion about these factors, see Ryder et al., 2015), it would be useful for future research to investigate the misinformation effect using a variety of perpetrator and misinformation faces. For example, future research could explore own-race bias in the context of misinformation and facial angles.

Like many other studies that adopt a lineup paradigm, a limitation of this research is the length of the distractor task – one minute. In real cases, the median average delay between witnessing a crime and being presented with a lineup is around 11 days in the United States (Flowe et al., 2018), and 31 days in the United Kingdom (Horry et al., 2012). Whilst this might seem concerning at face value, some studies have demonstrated that length of delay between encoding and test does not necessarily harm identification accuracy (Valentine et al., 2012; Wetmore et al., 2015). Nevertheless, other research finds that longer retention intervals are associated with decreased face recognition performance (Deffenbacher et al., 2008), and therefore, it would be valuable to investigate whether delay mediates the misinformation effect.

It might also be fruitful if future research considers whether a combined lineup procedure would have implications for these findings. That is, the lineup procedure at test could contain both the guilty suspect and misinformation suspect amongst fillers in a single lineup. A similar procedure has been used by some police departments, whereby everyone in the lineup is suspected of being the person (all-suspect design) who committed the offense (Wells and Luus, 1990). Whilst this lineup design has been used in forensic contexts, it is certainly not the norm and it would be unusual to have multiple suspects (i.e., one guilty and one innocent) in a single lineup. Nevertheless, it may be interesting for future research to explore this different method.

## 5 Conclusion

The impact of facial angle on recognition and discrimination accuracy was explored using a traditional misinformation paradigm (encoding, misinformation, test). Participants were not differentially likely to be misled by misinformation (i.e., an innocent suspect) depending on facial angle congruency across encoding, the misinformation, and lineup phases. This suggests that participants are no less likely to be misled if the innocent suspect’s face is presented in the same as opposed to different angle across encoding, misinformation, and test. Discrimination accuracy was significantly higher overall when the participants encoded the perpetrator from the front compared to the profile angle, suggesting that memory is stronger for faces that are originally encoded in frontal view. ROC analysis for all four conditions (FFF, PPP, FPF, PFP) also supported the encoding benefit of encoding a face from the front compared to the profile. Moreover, CAC analysis revealed a weak relationship between confidence and accuracy in the profile encoding (PPP and PFP) conditions compared to a stronger relationship in the frontal encoding (FFF and FPF) conditions. Given that legal decision makers rely on eyewitness confidence in court (Mickes, 2015; Garrett et al., 2020), they should be particularly aware that the reliability of eyewitness identifications could be impaired when a witness has encoded a perpetrator from a profile posed face (and discrimination accuracy is poor) compared to when the face is encoded from the front.

## Data availability statement

The datasets presented in this study can be found in online repositories. The names of the repository/repositories and accession number(s) can be found at: https://osf.io/fsmr9/ or https://osf.io/vdq63/.

## Ethics statement

The studies involving humans were approved by University of Birmingham STEM Ethics Committee. The studies were conducted in accordance with the local legislation and institutional requirements. The participants provided their written informed consent to participate in this study.

## Author contributions

KD, MC, and HF conceived of the idea and designed the experiments. KD collected the data. KD and MC analyzed the data. KD, TB, and HF wrote the paper with input from all authors. All authors contributed to the article and approved the submitted version.

## Footnotes

^1^ www.osf.io/5pr8n

^2^ https://github.com/E-Y-M/poweROC
